# Supplementary material for: Prevalence of Vitamin D Deficiency Among Adults in Kazakhstan: A Systematic Review and Meta-Analysis
Source: Medicina (Kaunas). 2024 Dec 11;60(12):2043. doi: 10.3390/medicina60122043 (PMC11727896; doi:10.3390/medicina60122043)
Supplement: Supplementary file 1 [file medicina-60-02043-s001.zip › medicina-3356179-supplementary-Table S1.pdf]

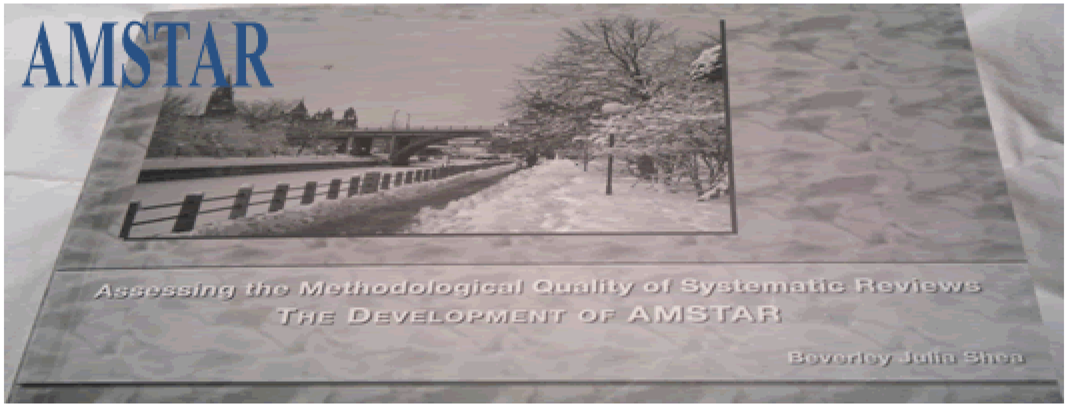

AMSTAR 2 Results

Printer Friendly Version

Article Name: Prevalence of Vitamin D Deficiency Among Adults in Ka-zakhstan: A Sys

---

Prevalence of Vitamin D Deficiency Among Adults in Ka-zakhstan: A Systemati is a High quality review

1. Did the research questions and inclusion criteria for the review include the components of PICO?

Yes
Yes
Yes
Yes
Yes

---

2. Did the report of the review contain an explicit statement that the review methods were established prior to the YesYesYesYesYesYesYesYes

**conduct of the review and did the report justify any significant deviations from the protocol?**	
**3. Did the review authors explain their selection of the study designs for inclusion in the review?**	Yes<br><br>Yes
**4. Did the review authors use a comprehensive literature search strategy?**	Yes<br>Yes<br>Yes<br>Yes<br><br>Yes<br><br><br>Yes
**5. Did the review authors perform study selection in duplicate?**	Yes<br>Yes
**6. Did the review authors perform data extraction in duplicate?**	Yes<br>Yes
**7. Did the review authors provide a list of excluded studies and justify the exclusions?**	Yes<br>Yes<br>Yes
**8. Did the review authors describe the included studies in adequate detail?**	Yes<br><br><br><br><br><br>Yes<br>Yes<br>Yes

	Yes<br>Yes
**9. Did the review authors use a satisfactory technique for assessing the risk of bias (RoB) in individual studies that were included in the review?**<br>**RCT**	
**NRSI**	Yes<br><br><br><br><br><br>Yes<br>Yes<br>Yes<br>Yes
**10. Did the review authors report on the sources of funding for the studies included in the review?**	No
**11. If meta-analysis was performed did the review authors use appropriate methods for statistical combination of results?**<br>**RCT**	
**NRSI**	Yes<br><br><br><br><br>Yes<br>Yes<br>Yes<br>Yes
**12. If meta-analysis was performed, did the review authors assess the potential impact of RoB in individual studies on the results of the meta-analysis or other evidence synthesis?**	Yes

**13. Did the review authors account for RoB in individual studies when interpreting/ discussing the results of the review?**	Yes<br>Yes
**14. Did the review authors provide a satisfactory explanation for, and discussion of, any heterogeneity observed in the results of the review?**	Yes<br>Yes
**15. If they performed quantitative synthesis did the review authors carry out an adequate investigation of publication bias (small study bias) and discuss its likely impact on the results of the review?**	Yes<br>Yes
**16. Did the review authors report any potential sources of conflict of interest, including any funding they received for conducting the review?**	Yes<br>Yes

To cite this tool: Shea BJ, Reeves BC, Wells G, Thuku M, Hamel C, Moran J, Moher D, Tugwell P, Welch V, Kristjansson E, Henry DA. AMSTAR 2: a critical appraisal tool for systematic reviews that include randomised or non-randomised studies of healthcare interventions, or both. BMJ. 2017 Sep 21;358:j4008.

<< Back

Copyright © 2024 AMSTAR All Rights Reserved |
